# Characteristics of prosthetic vision in rats with subretinal flat and pillar electrode arrays

**DOI:** 10.1088/1741-2552/ab34b3

**Published:** 2019-10-30

**Authors:** Elton Ho, Xin Lei, Thomas Flores, Henri Lorach, Tiffany Huang, Ludwig Galambos, Theodore Kamins, James Harris, Keith Mathieson, Daniel Palanker

**Affiliations:** 1 Department of Physics, Stanford University, Stanford, CA 94305, United States of America; 2 Hansen Experimental Physics Laboratory, Stanford University, Stanford, CA 94305, United States of America; 3 Department of Electrical Engineering, Stanford University, Stanford, CA 94305, United States of America; 4 Department of Applied Physics, Stanford University, Stanford, CA 94305, United States of America; 5 Institute of Photonics, University of Strathclyde, Glasgow, United Kingdom; 6 Department of Ophthalmology, Stanford University, Stanford, CA 94305, United States of America; 7 These authors contributed equally to this work.; 8 To whom correspondence should be addressed.

**Keywords:** retinal prosthesis, photovoltaics, restoration of sight, retinal degeneration

## Abstract

**Objective:**

Retinal prostheses aim to restore sight by electrically stimulating the surviving retinal neurons. In clinical trials of the current retinal implants, prosthetic visual acuity does not exceed 20/550. However, to provide meaningful restoration of central vision in patients blinded by age-related macular degeneration (AMD), prosthetic acuity should be at least 20/200, necessitating a pixel pitch of about 50 μm or lower. With such small pixels, stimulation thresholds are high due to limited penetration of electric field into tissue. Here, we address this challenge with our latest photovoltaic arrays and evaluate their performance *in vivo*.

**Approach:**

We fabricated photovoltaic arrays with 55 and 40 μm pixels (a) in flat geometry, and (b) with active electrodes on 10 μm tall pillars. The arrays were implanted subretinally into rats with degenerate retina. Stimulation thresholds and grating acuity were evaluated using measurements of the visually evoked potentials (VEP).

**Main results:**

With 55 μm pixels, we measured grating acuity of 48 ± 11 *μ*m, which matches the linear pixel pitch of the hexagonal array. This geometrically corresponds to a visual acuity of 20/192 in a human eye, matching the threshold of legal blindness in the US (20/200). With pillar electrodes, the irradiance threshold was nearly halved, and duration threshold reduced by more than three-fold, compared to flat pixels. With 40 *μ*m pixels, VEP was too low for reliable measurements of the grating acuity, even with pillar electrodes.

**Significance:**

While being helpful for treating a complete loss of sight, current prosthetic technologies are insufficient for addressing the leading cause of untreatable visual impairment—AMD. Subretinal photovoltaic arrays may provide sufficient visual acuity for restoration of central vision in patients blinded by AMD.

## Introduction

Age-related macular degeneration (AMD) is a leading cause of untreatable vision loss, affecting over 8.7% of the population worldwide [1]. Advanced forms of AMD (neovascularization and geographic atrophy) are associated with severe visual impairment, and their prevalence dramatically increases with age: from 1.5% in US population above 40 years to more than 15% in population older than 80 years [2]. Despite losing high-resolution central vision, these patients rarely exhibit visual acuity worse than 20/400 due to preservation of peripheral vision. Therefore, prosthetic restoration of sight in such conditions may only be beneficial if acuity reaches 20/200 or better.

In the healthy retina, photoreceptors convert light into electrical and chemical signals, which propagate to bipolar cells located in the inner nuclear layer (INL), and then to retinal ganglion cells (RGC), which generate trains of action potentials transmitted to the brain via the optic nerve. In retinal degenerative diseases, gradual loss of photoreceptors leads to visual impairment, while the remaining retinal neurons survive to a large extent [3–5].

Electronic retinal prostheses are designed to reintroduce visual information into the degenerate retina by electrical stimulation of the surviving inner retinal neurons. Current strategies involve placing electrode arrays either subretinally, to stimulate the first neural layer after photoreceptors (mainly bipolar cells in the INL) [6–8], or epiretinally, to target the output layer (RGCs) [9, 10].

Direct stimulation of RGCs with epiretinal implants bypasses the retinal network. With one action potential elicited by one stimulation pulse, theoretically, this approach may induce spike trains which reproduce the natural retinal code in each of the two dozen types of ganglion cells, if they could be identified and selectively stimulated [11]. However, the epiretinal implant currently approved for human use (ARGUS II, Second Sight Inc., Sylmar, California, USA) has electrodes much larger than cellular size (200 *μ*m in diameter, 575 *μ*m pitch)[12], which are relatively far (on average ~180 *μ*m) from the target cells [13] and result in indiscriminate activation of multiple cell types. Consequently, patients with this system reported extremely low visual acuity—no better than 20/1260 [14]. Moreover, epiretinal stimulation elicited responses not only from the underlying neurons, but also the bypassing axons from remote RGCs, causing distorted visual percepts [15].

On the other hand, bipolar cells can be modulated gradually, by amplitude or duration of the stimulus [16]. The elicited neural signals are then transmitted via retinal network to the ganglion cells, which respond with bursts of spikes. Such a network-mediated response of degenerate retina preserves many features of normal vision, including flicker fusion at high frequencies (>20 Hz)[17, 18], adaptation to static images [19], and antagonistic center-surround organization of receptive fields, as demonstrated with RCS rats [20].

In clinical trials of a subretinal implant (Alpha IMS/AMS, Retinal Implant AG, Reutlingen, Germany), visual acuity was typically below 20/1200, with two exceptional patients reaching 20/546 [21, 22]. This implant has 1500 to 1600 pixels, each 70 *μ*m in size. Since the theoretical limit of resolution with this array is about 20/280, it is alarming that none of the patients achieved such levels of acuity. One reason could be due to the monopolar design of this implant, where active electrodes in each pixel share a common remote return electrode. This results in strong cross-talk between the neighboring electrodes, leading to greatly reduced spatial contrast [23].

To reduce the cross-talk, we have improved the localization of electric field to the level required for higher visual acuity by developing a photovoltaic subretinal prosthesis with active and return electrodes in each pixel. Photodiodes convert pulsed light projected from augmented reality glasses [24] into electric current that flows through the tissue between two electrodes in each pixel, stimulating the nearby inner retinal neurons—mostly bipolar cells [8, 25]. To avoid visual perception of bright light by remaining photoreceptors, we use near-infrared (NIR, 880–915 nm) wavelengths. Direct photovoltaic conversion of light into electric current eliminates the need for power supply and cables, which greatly simplifies surgical procedures and reduces associated postoperative complications [26].

Previously, we demonstrated that implants with 75 *μ*m pixels provided grating acuity matching the pixel pitch in rats [18]. Here, we show that subretinal pixels can be miniaturized further, while still eliciting retinal response well within the safety limits. Grating acuity with these arrays matches the pixel pitch below 50 *μ*m, corresponding to the threshold of legal blindness (20/200) in the US.

**Figure 1.**
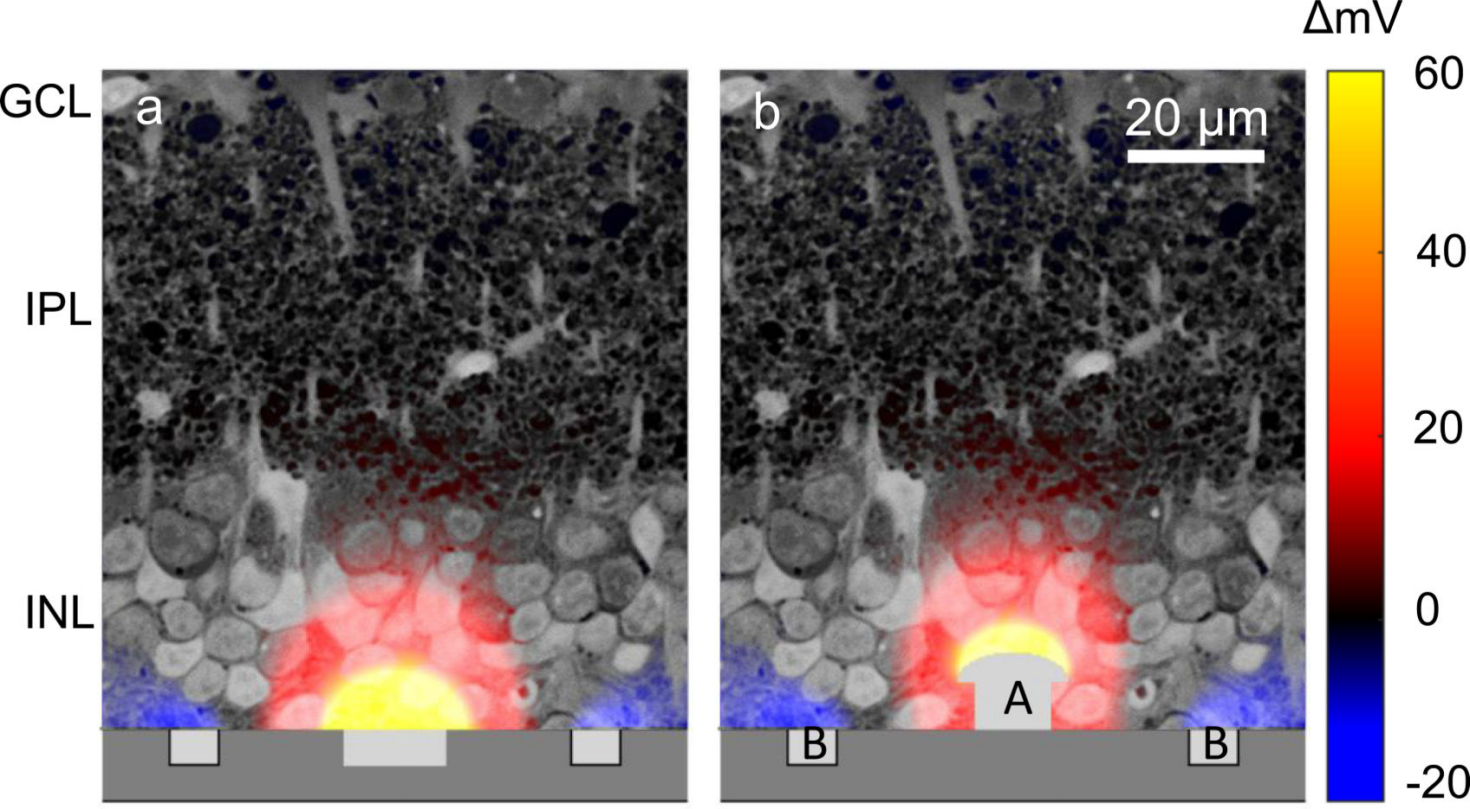
Illustration of electric potential with 55 *μ*m-pixel implants using a previously described model [27], plotted over a histological image of the rat retina. (a) With flat pixels, the top cells in the inner nuclear layer (INL) are not stimulated. (b) By elevating the active electrode [1] halfway into the INL, electric field can penetrate deeper into the INL. Return electrode [2] remains on the surface of the device.

One of the major problems with reducing the size of bipolar pixels is that this miniaturization decreases penetration depth of electric field into tissue (figure 1). Also, smaller electrodes have lower charge injection capabilities. To deliver electric field closer to the target cells, we fabricated devices with active electrodes elevated on top of pillars [27]. After implantation, cells of the INL migrated into the space between the pillars, improving the proximity of 3D electrodes to neurons, which led to reduction in stimulation thresholds. Not only does this enable safer activation of cells, but it also widens the dynamic range of prosthetic vision for better encoding of the visual information.

## Methods

### Implant fabrication

**Figure 2.**
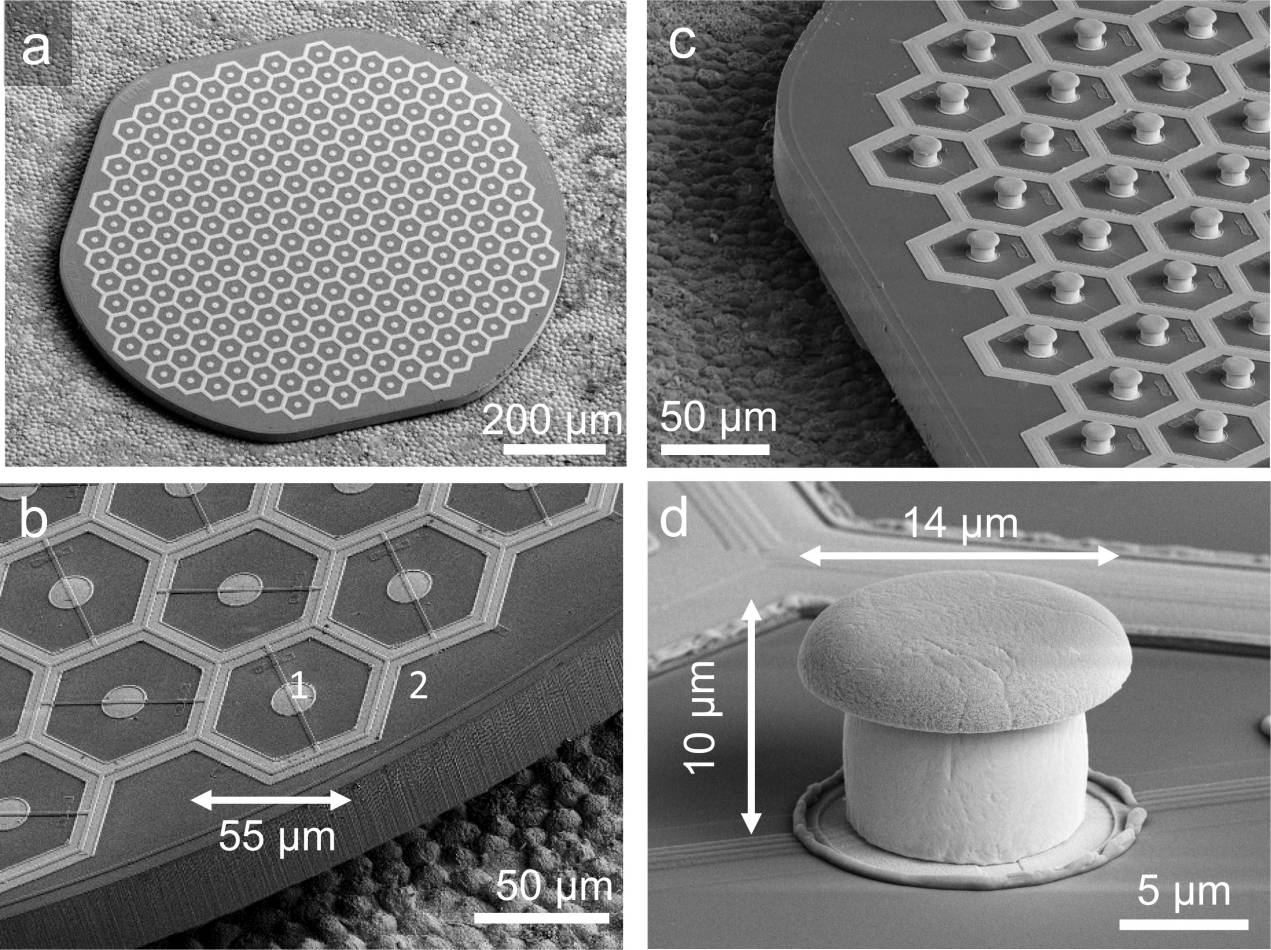
Scanning electron micrographs (SEM) of the hexagonal photovoltaic arrays with 55 *μ*m pixels. (a) The whole implant of 1 mm in width, containing 250 pixels. The array was placed on top of the RPE for scale. (b) Higher magnification of the implant demonstrates relative sizes of the central active electrode [1] and circumferential return electrode [2] in flat pixels. The active electrode is 14 *μ*m in diameter, and return electrodes are 9 *μ*m wide. (c) Similar array with pillar electrodes. (d) Image of a single pillar electrode with a SIROF-coated cap. The pillar is 10 *μ*m in height, with a cap width of 14 *μ*m and stem width of 10 *μ*m.

The photovoltaic arrays were designed based on the fabrication and operation principles published earlier [27, 28], using an updated fabrication process and smaller pixel sizes. Implants of 1 mm in diameter and 30 *μ*m in thickness consist of hexagonally arranged photovoltaic pixels (figure 2). In a rat eye, these implants cover approximately 20° of the visual field [29]. In the current study, pixels were either 40 or 55 *μ*m in width, corresponding to 502 or 250 pixels in each array, respectively. Due to the hexagonal arrangement, spacing of the adjacent rows, i.e. pixel pitch, is 35 and 48 *μ*m, respectively. Each pixel includes two diodes connected in series between the active (A) and return electrode (B) (figure 2) to maximize the efficiency of subretinal charge injection and stimulation [30]. The diodes are fabricated on n- silicon substrate (phosphorus 10^15^ cm^−3^) with p+ doping (boron 10^19^ cm^−3^) and a junction depth of 1.5 *μ*m. Active electrodes are connected to the p+ regions, so that they develop a positive potential with respect to return electrode when the device is illuminated. To increase the photosensitive area compared to previous design [18], we minimized the width of the isolation trenches between diodes and between pixels to 1 *μ*m. We also eliminated the 5 *μ*m-wide open trenches between pixels, which were helpful in the previous implants for diffusion of oxygen and nutrients in *ex vivo* experiments but are not required *in vivo* due to presence of the retinal vasculature. Return electrodes connected across the entire array are shared across the pixel boundaries, and thereby cover the isolation trenches between pixels. Active electrodes are 10 and 14 *μ*m in diameter, and the width of the shared return electrode is 6 and 9 *μ*m for 40 and 55 *μ*m pixels, respectively (i.e. 3 and 4.5 *μ*m per pixel), so that the area of the returns is about five times that of the active electrodes (figure 2(b)). Active and return electrodes were coated with sputtered iridium oxide film (SIROF) to create a high-capacitance electrode–electrolyte interface. To prevent the implant erosion and provide an antireflection coating, all implants were covered with 380 nm of amorphous silicon carbide (SiC) on top of 70 nm of silicon dioxide (SiO_2_), optimized for 880 nm illumination [31].

Pillars were electroplated with gold on top of the photovoltaic pixels to a height of 10 *μ*m, with hemispherical caps extending to 10 and 14 *μ*m in diameter for 40 and 55 *μ*m pixels, respectively (figure 2(d)). Current density in steady state is proportional to capacitance per unit area [32]. Since the SIROF capacitance is about 100 times higher than that of gold (1 mF cm^−2^ versus 0.01 mF cm^−2^) [30, 33], and the area of a gold pillar is similar to that of the SIROF cap, current flows predominantly through the SIROF-coated cap, even though the pillar sidewalls are not insulated. Electroplating was performed through a patterned photoresist mold [27] using a sacrificial aluminum (Al) layer to connect the active electrodes to the current source.

For the rest of this paper, we will use the following nomenclature: F55 and F40 for flat arrays with 55 and 40 *μ*m pixels, respectively, and Pil55 and Pil40 for arrays with pillar electrodes of the same pixel sizes.

### Animals and implantation

Royal college of surgeons (RCS) rats were used as an animal model of inherited retinal degeneration. In these animals, a mutation in the MERTK gene reduces the phagocytic capability of the retinal pigmented epithelium (RPE), leading to degeneration of photoreceptors by four months [34]. Rats were implanted after the loss of photoreceptors, and the follow-up continued for the life of the animals (up to one year). The animals were housed and maintained at the Stanford animal facility with a 12 h light/12 h dark cycle with food and water ad libitum. Adult Long-Evans WT rats were purchased from Charles River Laboratories (Wilmington, MA, USA) as a wild-type control for measurements of the grating acuity (*n* = 6) and frequency response (*n* = 5). All *in vivo* experimental procedures were conducted in accordance with the Stanford University institutional guidelines and conformed to the guidelines of the Association for Research in Vision and Ophthalmology (ARVO) Statement for the Use of Animals in Ophthalmic and Vision research.

A total of 20 animals were implanted with F55 (*n* = 5), Pil55 (*n* = 5), F40 (*n* = 5), and Pil40 (*n* = 5). The subretinal implantation technique was similar to the one previously reported by our group [18]. Animals were anaesthetized with a cocktail of ketamine (75 mg kg^−1^) and xylazine (5 mg kg^−1^) injected either intraperitoneally or intramuscularly. A 1.5 mm incision was made through the sclera and choroid 1.5 mm posterior to the limbus, and the retina was lifted with an injection of saline solution. For pillar arrays, a viscoelastic solution (Viscoat, sodium chondroitin sulfate 4%-sodium hyaluronate 3%) was dropped on top of the implant to prevent pillars from catching onto the retina during insertion. Upon insertion of the array into the subretinal space, the sclera and conjunctiva were sutured with nylon 10–0, and topical antibiotic (Bacitracin/Polymyxin B) applied on the eye postoperatively. Surgical success and retinal reattachment were verified using optical coherence tomography (OCT) (HRA2-Spectralis; Heidelberg Engineering, Heidelberg, Germany) immediately after surgery. The retina detached during surgery settled onto flat implants within a week post-surgery, similarly to our previous studies with larger pixels [18]. Based on our previous anatomical studies with pillar arrays [27], we allowed six weeks post implantation for pillar integration with the retina. We inspected implant stability with OCT again before VEP measurements. All implants remained stable in the subretinal space throughout the follow-up period, lasting up to a year.

Three transcranial screw electrodes (00 × ¼ʺ stainless steel, part FF00CE250; Morris, Southbridge, MA, USA) were implanted and secured in place with cyanoacrylate glue and dental acrylic. The electrodes penetrate only the skull but not the brain tissue. One electrode was placed at each hemisphere of the V1 visual cortex (4 mm lateral from midline, 6 mm caudal to bregma), and a reference electrode was placed 2 mm right of midline and 2 mm anterior to bregma. Nose and tail needle electrodes served as a reference and ground, respectively (supp. figure 1(b) (stacks.iop.org/JNE/16/066027/mmedia)).

### Retinal stimulation

Rats were anesthetized with a cocktail of ketamine (37.5 mg kg^−1^) and xylazine (2.5 mg kg^−1^) injected intramuscularly. Steady anesthesia was maintained using the following measures: periodic monitoring of spontaneous eye movements and respiratory patters; supplementary injection of half the initial dose every 40 min, or as needed.

Near-infrared (NIR, 915 nm) and green (532 nm) lasers from single-mode fibers were collimated and patterned using a digital micromirror display (DMD; DLP Light Commander; LOGIC PD, Carlsbad, CA, USA). The optical system was mounted on a slit lamp (Zeiss SL-120; Carl Zeiss, Thornwood, NY, USA) to allow direct observation of the patterns on the retina with a NIR-sensitive CCD camera (acA1300–60gmNIR; Basler, Ahrensburg, Germany) (supp. figure 1(a)). Following pupil dilation (and ocular retraction in some cases), the cornea was covered with a viscoelastic gel and a cover slip to cancel the optical power of the eye and ensure good retinal visibility. The posture of the animal was adjusted to ensure normal beam incidence on the implant center. For fullfield measurements, NIR stimulation was applied with pulse durations ranging from 0.06 to 10 ms, peak irradiances from 0.06 to 8 mW mm^−2^, and frequencies from 2 to 64 Hz. Linear grating patterns, ranging from 10 to 240 *μ*m per stripe, were generated with a custom software. Gratings were alternated (contrast reversal) at 1 Hz, while the light sources were pulsed at 40 Hz using 4 ms flashes at 8 mW mm^−2^ and 100 nW mm^−2^ for 915 nm and 532 nm wavelengths, respectively. Stimulation parameters are listed in table 1. As a control, we applied NIR pulses (2 Hz, 10 ms) at 8 mW mm^−2^ on a 1 × 1 mm^2^ area outside the implant to ensure that there was no photoreceptormediated response.

### Visually-evoked potentials (VEP) recording and analysis

VEP were recorded using the Espion E2 system (Diagnosys, Lowell, MA, USA) at 1 kHz sampling rate using a 0.5–500 Hz bandpass filter and averaged over 500 trials for each experiment. The VEP amplitude was quantified as the peak-to-peak voltage of the signal within 350 ms post stimulus. A detectable VEP response was defined as a deviation from the baseline by more than six times the noise level, determined as RMS (s.d.) of the signal during 50 ms preceding the stimulus, similar to our previous studies with larger pixels [18]. In addition, we applied an unpaired *t*-test to compare the VEP amplitude at a given stimulus parameter to that at the noise level in the population of test animals. Modulation of the VEP amplitude by light intensity (with 10 ms pulses) and by pulse duration (at 8 mW mm^−2^) was plotted normalized to the noise amplitude in each animal (example traces in supp. figure 1(c)). All electrophysiological measurements were conducted during the months 1–12 post implantation.

### Visual acuity measurements

Visual acuity was assessed by recording the cortical response to alternating gratings of various spatial frequencies, as described previously [18, 35]. For natural acuity measurements, WT rats (*n* = 6) were shown gratings with the stripe width ranging from 10 to 240 *μ*m, delivered at 1 Hz reversal rate and 40 Hz carrier frequency. For accurate assessment of the noise level, we applied a 40 Hz notch filter to remove oscillations due to the flicker, which are more pronounced at high spatial frequencies. The VEP amplitude was defined as the peak-to-peak voltage of the cortical signal during the first 350 ms post stimulus. For NIR stimulation, stripe widths varied from 20 to 240 *μ*m (*n* = 5). For various grating sizes, VEP amplitude was normalized to the maximum in each animal, and the noise level was defined as the amplitude at the smallest grating size (example traces in supp. figure 1(d)). To define the acuity, the averaged VEP amplitude was plotted as a function of the stripe width and fit with a 2nd-degree polynomial function using the 20, 40, 60, 80 *μ*m data points for visible light and the 50, 55, 60, 80, 120 *μ*m data points for prosthetic stimulation. The visual acuity limit was defined as the intersection point of the fitted curve with the noise level. We also tried curve fitting with polynomials of other degrees and including different data points, which resulted in lower estimates for the smallest resolvable gratings, i.e. higher grating acuity. The chosen fit yielded the most conservative estimate.

## Results

### Stimulation thresholds

**Figure 3.**
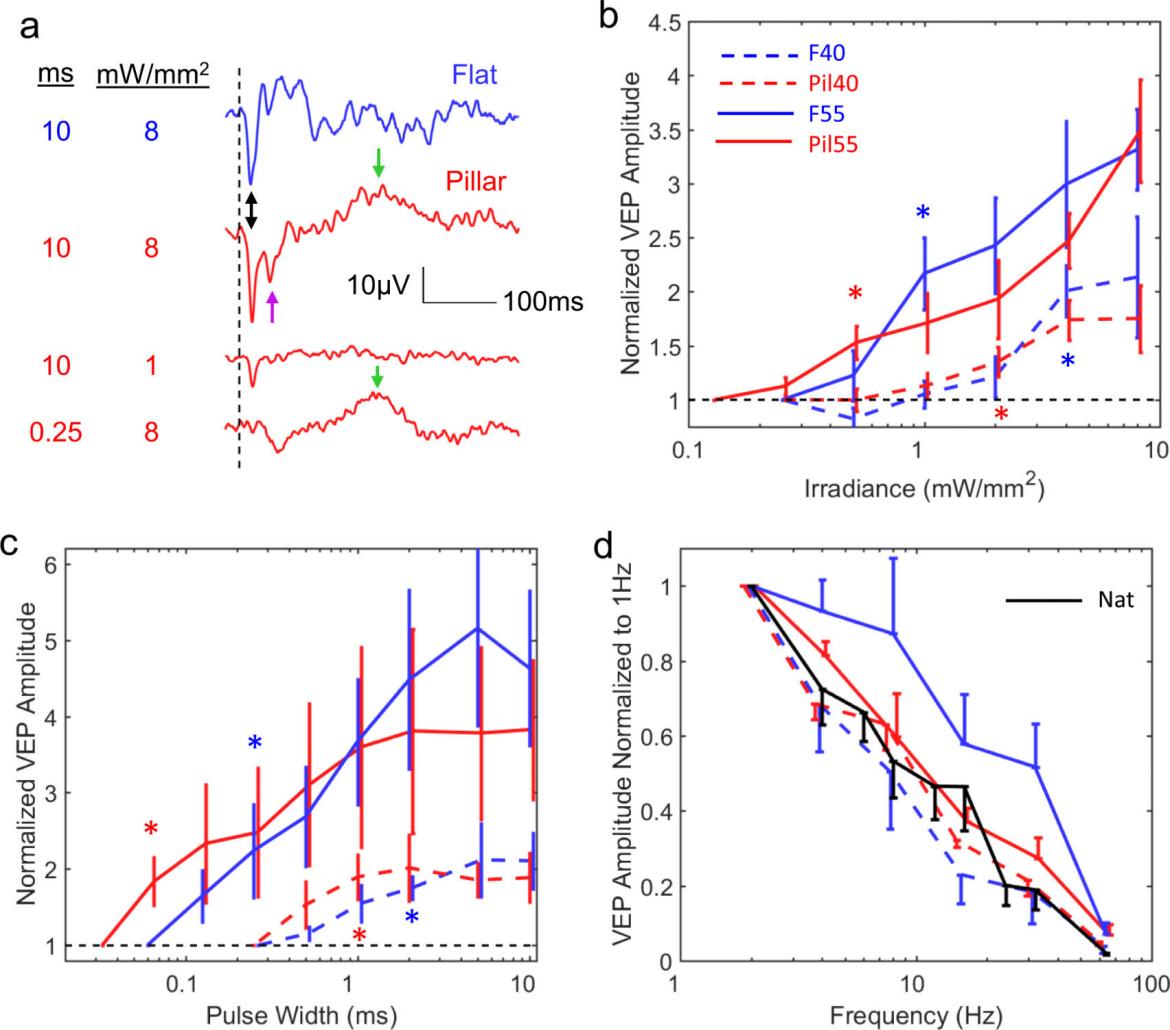
Visually evoked potentials (VEP) and stimulation thresholds. (a) Example VEP waveforms with flat and pillar 55 *μ*m implants at various irradiances and pulse durations. The traces were averaged over 500 trials. The double-headed arrow indicates the primary peak located at ~17 ms post stimulus. The purple arrow indicates the secondary negative peak that has high irradiance threshold. The green arrows indicate a VEP component that is highly sensitive to pulse duration but not irradiance. (b) Variation of the VEP amplitudes with irradiance. Stars indicate the lowest irradiance at which *p* < 0.05 (unpaired *t*-test, *n* = 5 for each implant type). Thresholds are summarized in table 1. (c) Variation of the VEP amplitude with pulse width. Stars indicate the shortest duration at which *p* < 0.05 (same as (b)). Thresholds are summarized in table 2. (d) Variation of the VEP amplitude with frequency for all 4 implant types and for normal vision (*n* = 5). For the plot clarity, we used one-sided error bars offset horizontally by the line width in order to avoid overlapping with adjacent bars. All error bars are shown in terms of s.e.m.

Response to prosthetic stimulation was evaluated by recording VEP via transcranial electrodes placed above the visual cortex, as described previously [18, 25] and exemplified in figure 3(a). A near-infrared beam (915 nm) reflected off the DMD, was projected onto the implant from a slit lamp. Stimulation thresholds with respect to irradiance and pulse duration, as well as variation of the VEP amplitude with frequency, were measured in the ranges summarized in table 1 (see methods). The VEP amplitude was quantified as the peak-to-peak voltage of the recording within 350 ms post stimulus, and an amplitude greater than six times the RMS noise was considered a signal above threshold. Previous experiments demonstrated that VEP is not present when conduction along the optic nerve is blocked [25]. We also verified that RCS rats do not respond to NIR flashes projected outside the implant.

F55 implants (*n* = 5) induced cortical response above 1.0 ± 0.27 mW mm^−2^ (s.e.m.), while with Pil55 implants (*n* = 5) the threshold was 0.55 ± 0.15 mW mm^−2^ (figure 3(b)). The 45% decrease in stimulation threshold agrees with our previous modeling results [27]. With increasing irradiance, the cortical response with flat implants maintained generally the same shape (figure 3(a)), while its amplitude increased with irradiance (figures 3(b) and supp. figure 2). Signals with pillar implants had a distinctly different shape: in addition to a short-latency negative peak at ~20 ms (doubleheaded arrow in figure 3(a)), there was a second negative peak at ~40 ms and a positive peak about 100 to 200 ms later. The threshold of the second negative peak (purple arrow) was approximately an order of magnitude higher than that of the first negative peak.

The effect of pillars was much more pronounced on the threshold pulse duration: it decreased by 78%—from 0.29 ± 0.11 ms (s.e.m.) for F55 implants to 0.08 ± 0.02 ms for Pil55 arrays (figure 3(c)). The VEP of flat implants maintained the same shape as pulse duration varied, so that only the short (~20 ms) negative wave was detectable near the threshold. However, with pillars, the negative peak at 20 ms disappeared for very short pulses (<0.25 ms), while the much later positive component remained prominent (figure 3(a) green arrow).

Previous studies demonstrated that in healthy retina responding to pulsed visible light *ex vivo*, flicker fusion occurs at lower frequencies than in degenerate retina responding to prosthetic stimulation with 70 *μ*m pixels [18]. Our current measurements *in vivo* confirmed this effect for 55 *μ*m flat implants, with the normalized VEP amplitude of prosthetic vision at 20 Hz being about twice that of natural, and reaching the same level beyond 50 Hz (figure 3(d)). Slower decline of retinal response with increasing frequency in prosthetic vision is likely due to absence of photoreceptors, the photochemical processes of which are much slower than the rest of the retinal network. However, with all pillar arrays and with 40 *μ*m flat pixels, retinal response declined with frequency as fast as that of natural vision, suggesting another potential difference in the retinal stimulation mechanisms.

With F40 arrays (*n* = 5), the thresholds were significantly higher (1.8 ± 0.58 mW mm^−2^ and 0.83 ± 0.17 ms) and the maximum VEP amplitude about twice lower than that with 55 *μ*m pixels (figure 3(b)). Even though the threshold is below the ocular safety limit (5 mW mm^−2^ average irradiance at 880 nm [36]), not much range remains for encoding grey levels and assessing grating acuity, which requires a good signal-to-noise ratio achieved at irradiance levels far above the stimulation threshold. Pil40 arrays (*n* = 5) had thresholds of 1.3 ± 0.27 mW mm^−2^ and 0.7 ± 0.12 ms, but this improvement did not result in increase of the maximum VEP amplitude—it was still only half that with 55 *μ*m pixels. The effect of pillars on pulse duration with 40 *μ*m pixels was also much smaller than with 55 *μ*m.

### Grating acuity

**Figure 4.**
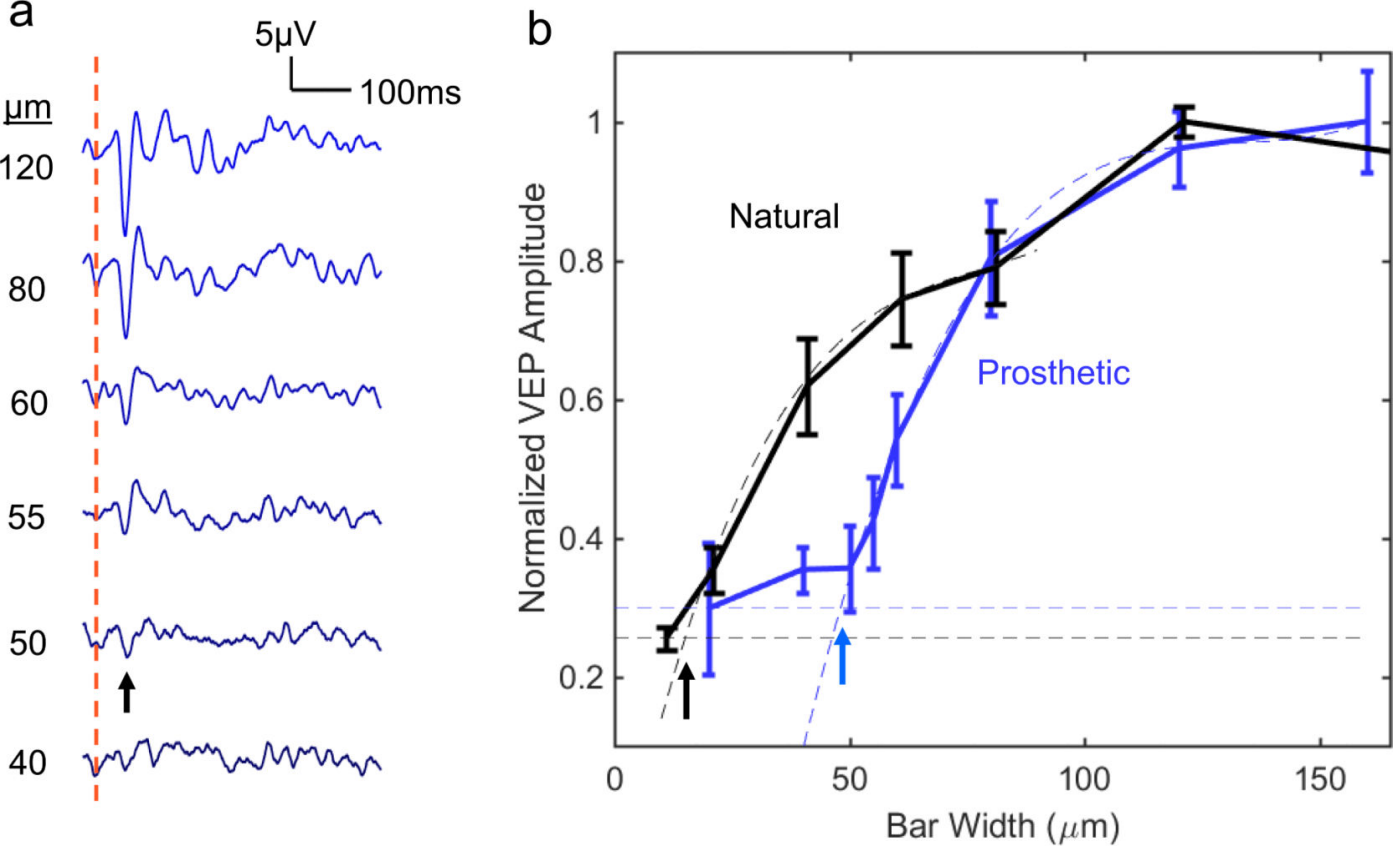
Grating acuity. (a) Averaged prosthetic VEP response to alternating gratings with 55 *μ*m pixels (*n* = 5). The red dash line indicates the instance of the grating reversal. (b) Prosthetic and natural VEP amplitude as a function of the grating stripe width. Smaller stripe width corresponds to higher grating acuity. Acuity limit, defined as the intersection of the fitting line with the noise level (horizontal dash lines), is 48 ± 11 *μ*m for prosthetic response, and 17 ± 5 *μ*m for natural vision. All errors are listed in terms of s.e.m.

Measuring the cortical response to alternating gratings is an established method to assess visual acuity in animals [35] and in human infants [37, 38]. Visual acuity measured with this method matches that of behavioral tests [39]. We recorded the VEP response to alternating grating patterns projected onto the implant with 55 *μ*m pixels in RCS rats. Images were delivered with NIR light at 8 mW mm^−2^ peak irradiance using 4 ms pulses at 40 Hz repetition rate, and pattern reversal at 1 Hz. The resulting VEP waveforms contained both a 2 Hz (pattern reversal-induced) and 40 Hz (pulse-induced) component. Using a 40 Hz notch filter, we singled out the pattern reversal-induced response (figure 4(a)), with its amplitude measured as the peak-to-peak voltage between 0 and 100 ms after each pattern reversal. As a control, the same experiment was performed on healthy rats (Long Evans, *n* = 6) using green light (532 nm) illumination pulsed at 40 Hz.

The grating acuity limit was assessed by extrapolating the measured data down to noise level [18] (see methods). Smaller grating width corresponds to better grating acuity. As can be seen in figure 4(b), for prosthetic vision with 55 *μ*m pixels, this limit corresponds to 48 ± 11 *μ*m (s.e.m.). In a hexagonal array, adjacent rows are separated by *w* = *d*=·cos(30 °) =*d*√3/2 = 0.87*d*, where *d* is the pixel width. For *d* = 55*μ*m the distance between adjacent rows of pixels is *w* = 48*μ*m, matching the measured acuity. For natural vision, the measured grating acuity limit is 17 ± 5*μ*m. With 40*μ*m pixels, even having pillar electrodes, the VEP amplitude was too low for a reliable measurement of the grating acuity.

In measurements of the grating acuity, there is a concern whether the detected VEP response resulted from aliasing or truly resolving the grating. According to the Nyquist sampling theory, spatial resolution (minimum stripe width of the grating) of the sensor array with a pixel size *d*, is limited by the row pitch, which for a hexagonal array is 0.87*d*. To assess the extent of aliasing, we simulated the pixel activation pattern when a grating image is projected onto a hexagonal array. As shown in supplemental figure 3, for grating widths larger than 0.8*d*, the pixelated image matches the original pattern. As the bar width decreased to approximately 0.7*d*, the orientation of the pixelated image became ambiguous. With 55 *μ*m pixels, 0.7*d*= 38.5*μ*m. As can be seen in figure 4, prosthetic VEP in our measurements did not drop to the noise level at 40 *μ*m, indicating that aliasing may be involved in this response. To avoid any potential effect of aliasing on assessment of the grating acuity, we did not include any data points below the sampling density limit into the extrapolation dataset.

## Discussion

Our results demonstrate that hexagonal photovoltaic arrays with 55 *μ*m pixels provide a grating acuity matching the minimum distance between adjacent rows, i.e. the Nyquist sampling limit of 48 *μ*m. Depending on the orientation of the grating, visual acuity with such arrays ranges from 20/192 to 20/220 in a human eye. If successful in human trials, prosthetic vision with such spatial resolution should benefit not only the patients blinded completely by inherited retinal degeneration (such as Retinitis Pigmentosa), but also much more patients with central vision loss due to advanced AMD.

Although the retinal circuitry undergoes drastic remodeling during the end-stage of degeneration, when all photoreceptors are lost, as in Retinitis Pigmentosa [40, 41], recent clinical trials have demonstrated shape perception with subretinal electrical stimulation in RP patients [22]. In AMD patients, photoreceptors are lost only within a few-mm-wide zone in the central macula, and the inner retinal structure is much better preserved in these areas, compared to the end-stage of RP. Therefore, restoration of central vision in AMD patients with subretinal implants might provide even better results, as evidenced by the recent success of the PRIMA implant [42]. However, retinal degeneration may still limit the attainable visual acuity, and this effect remains to be tested with high resolution implants in clinical trials.

Stimulation threshold of subretinal implants increases with decreasing size *d* of bipolar pixels approximately as *1*/*d*^2^: from 0.13 to 0.55, 1.0, and 1.8 mW mm^−2^ with pixels of 140, 70, 55, and 40 *μ*m in size [17, 18]. This is largely due to the fact that the electric field penetrates into the tissue by approximately half a pixel width. Pillar electrodes improve proximity to target neurons, and therefore can reduce the stimulation threshold to some extent. However, they do not allow a very significant decrease in pixel size since this design is still limited by the geometry of spherical expansion of electric field.

Surprisingly, pillar electrodes affected the shape of the visually evoked potential and its dependence on pulse duration. In particular, pillars reduced the pulse duration threshold more than three-fold, when compared to flat arrays. Since the ocular safety limit is set primarily by cumulative heating [36], reduced pulse duration helps in this regard nearly as much as reduced irradiance.

However, with 40 *μ*m pixels, not only was the stimulation threshold nearly tripled, but also the maximum VEP response was halved, when compared with 55 *μ*m arrays. Even with pillar electrodes, the SNR was too low for acuity measurements, and we could not take full advantage of the reduced pulse duration threshold by increasing the pulse amplitude due to limited peak brightness of the beam. Therefore, other geometries should be explored for improving stimulation efficacy and further reduction (beyond 40 *μ*m) of the pixel size [43, 44].

Currently, it is not clear why the shape of the VEP signal elicited by pillar electrodes is different compared to planar implants, or why it changes with irradiance and with pulse duration. It could be due to some discrimination between the cell types residing at different depths of the INL [45] or due to heterogeneous distribution of ion channels [46], which play a more prominent role in non-monotonic fields [16]. It will be interesting to see whether these differences will affect the visual percepts in clinical testing.

In conclusion, in rats with retinal degeneration, hexagonal arrays with 55 *μ*m pixels provide grating acuity matching the row spacing of 48 *μ*m, which in a human eye geometrically corresponds to visual acuity matching the threshold of legal blindness (20/200). If successful in clinical testing, such arrays could provide highly functional prosthetic vision even for patients with the loss of only central vision, as in AMD. Scaling the pixel size further down is difficult even with pillar electrodes since stimulation thresholds approach the ocular safety limit and the cortical signal becomes too weak for electrophysiological measurements.

## Supplementary Material

supplementary

## Figures and Tables

**Table 1.** Ranges of the stimulation parameters in various measurements.

	Irradiance (mW mm^−2^)	Pulse duration (ms)	Repetition rate (Hz)

Irradiance threshold	0.125–8	10	2
Pulse duration threshold	8	0.03–10	2
Frequency variation	8	4	2–64

**Table 2.** Stimulation thresholds with 4 types of implants. All errors are listed in terms of s.e.m.

Implant type	F55	Pil55	F40	Pil40

Irradiance threshold (mW mm^−2^)	1.0 ± 0.27	0.55 ± 0.15	1.8 ± 0.58	1.3 ± 0.27
Duration threshold (ms)	0.29 ± 0.11	0.08 ± 0.02	0.83 ± 0.17	0.7 ± 0.12
