# Supplementary material for: Characteristics of prosthetic vision in rats with subretinal flat and pillar electrode arrays
Source: J Neural Eng. Author manuscript; Available in PMC 2020 Apr 30. (PMC7192047; doi:10.1088/1741-2552/ab34b3)

Supplemental Material

Aliasing

In measurements of the grating acuity, there is a concern whether the detected VEP response resulted from aliasing or truly resolving the grating. According to the Nyquist sampling theory, spatial resolution (minimum stripe width of the grating) of the sensor array is limited by the row pitch, which for a hexagonal array is *0.87d*. To assess the extent of aliasing, we simulated the pixel activation pattern when a grating image is projected onto a hexagonal array. This is quantified by applying an image similarity test between grating images and their corresponding implant activation maps. Activation maps were created by overlaying a grating image on top of a hexagonal sensor array, and then assigning a value between 0 and 1 (rounded to the nearest 1/8) to each hexagon proportional to the illuminated area in each photodiode. The 8 levels of grey were selected based on 12% contrast sensitivity of prosthetic vision previously measured in rats (48). The resulting activation map was then compared with gratings of different orientations by taking the Euclidean Distance (ED) defined as

$$ED = \sqrt{\sum_i (x_i - g_i)^2}$$

where x_i and g_i are values of the i-th pixel in the activation map and grating image, respectively, and i is summed over the whole image. This process was performed for a multitude of grating stripe widths. Intuitively, small ED means high resemblance.

Supplemental Figure 3 demonstrates the ED between the original grating and the images sampled by the array for one particular orientation centered at 0 rad. For grating widths larger than *0.8d*, the minimal ED was always found at 0 rad, indicating that pixelated image matches the original pattern. As the bar width decreased to approximately *0.7d*, the orientation of the pixelated image became ambiguous, exhibiting the ED minima not only at correct orientation, but also at another rotational angle (indicated by the arrow in Figure 5).

Supplemental Figure 1. Illustration of the experimental set-up and data analysis. (a) Schematic of the image projection system and an example recording (averaged over 500 trials). The VEP amplitude was taken as the peak-to-peak reading between 0 to 350 ms post-stimulus. (b) Electrode mapping. The recording electrode was placed at the V1 visual cortex contralateral to the implanted eye. Different reference electrodes were used for different channels: (1) nose, (2) 2 mm anterior to bregma and 2 mm right of midline, and (3) V1 visual cortex ipsilateral to the implanted eye. All channels were grounded to the tail. In this study, channel 3 yielded the strongest signals. (c) Example F55 VEPs in responses to 8 mW/mm^2 10-ms pulses. Dashed line indicates the onset of the stimulus. With a single trial, VEP was not discernable from the noise, while after averaging over 500 trials the signal becomes very clear. (d) Example of the F55 VEPs for alternating gratings. Dashed line indicates the timing of pattern alternation. After 500 trials, VEPs of the two phases of the grating cycle were summed together to compute the total VEP response, shown in Figure 4a.

Supplemental Figure 2. The VEP waveforms measured with F55 implants as a function of (a) irradiance and (b) pulse duration.

Supplemental Figure 3. Simulation of aliasing with the implant. (a) Euclidean distance (a.u.) between the implant activation map and the projected image, as a function of the normalized grating size and orientation. The black dash line indicates the actual orientation of the source grating image. Low values imply high image similarity. (b) Cross-sections at 1.5 and 0.7 pixel size. Black arrows point at the correct orientation (centered at 0

rad), and the grey arrow indicates aliasing. For grating stripe widths above 0.8 pixel size (e.g. top panel and green dash plane in (a)), there is only one significant “dip”, indicating high confidence in predicting the correct grating orientation. At around 0.7 pixel size (lower panel and blue dash plane in (a)) there are two dips (black and grey dash line), one of which represents aliasing.

Supplemental Figure 1

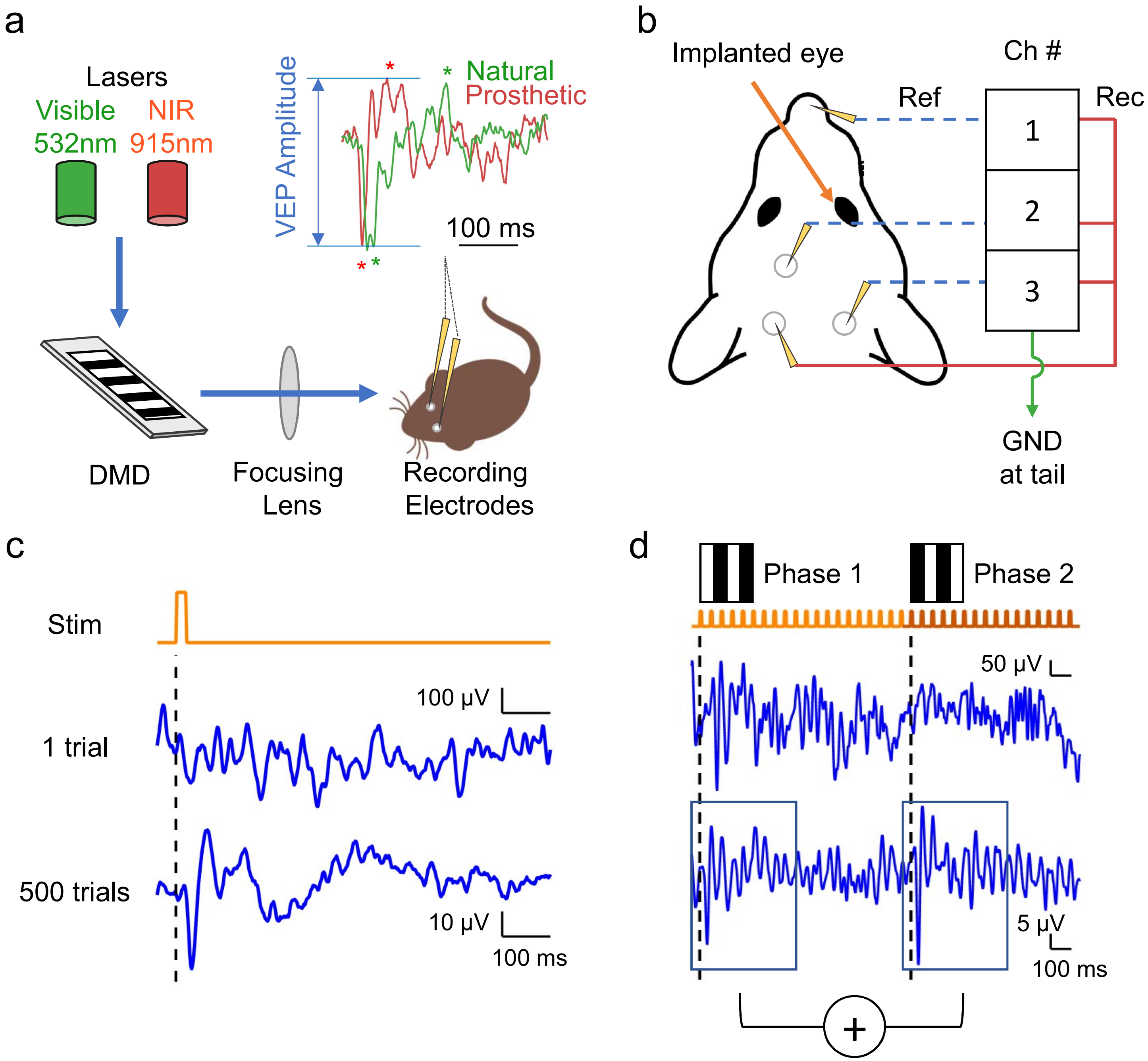

Supplemental Figure 2

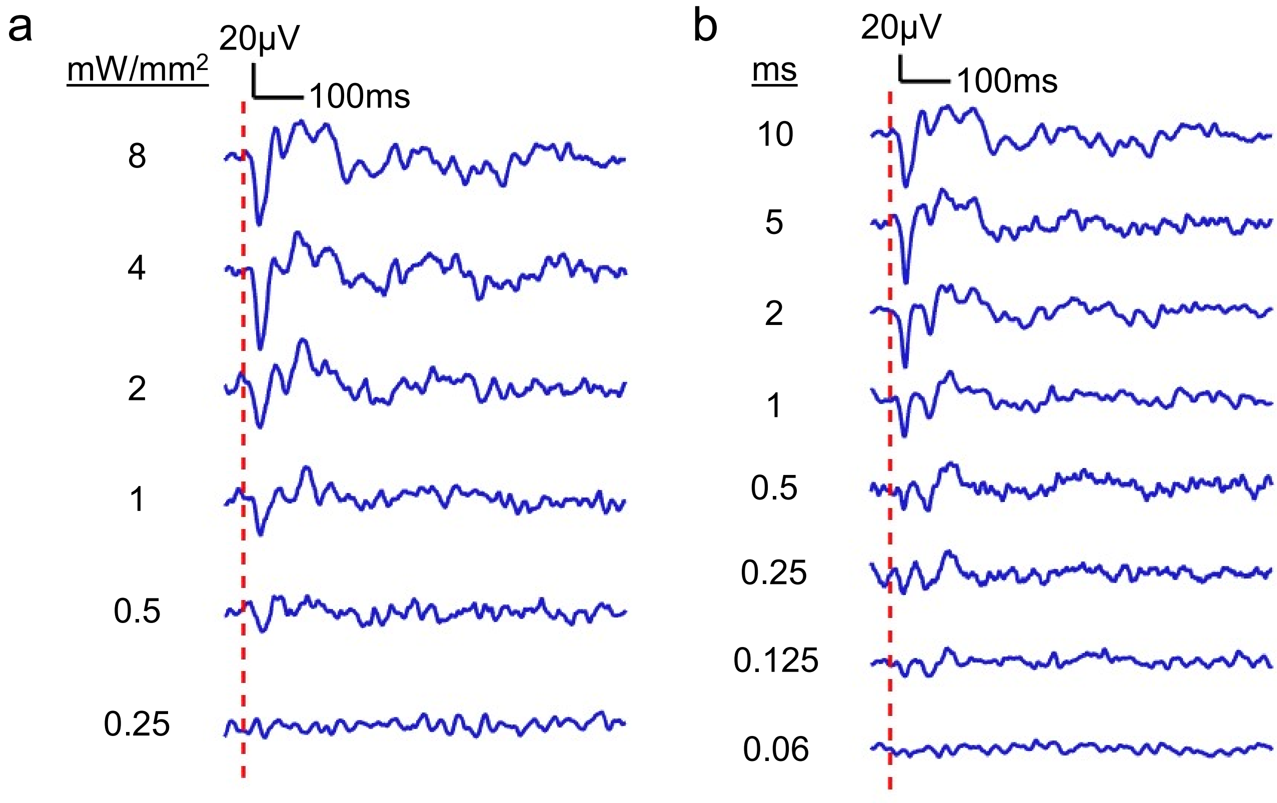

Supplemental Figure 3

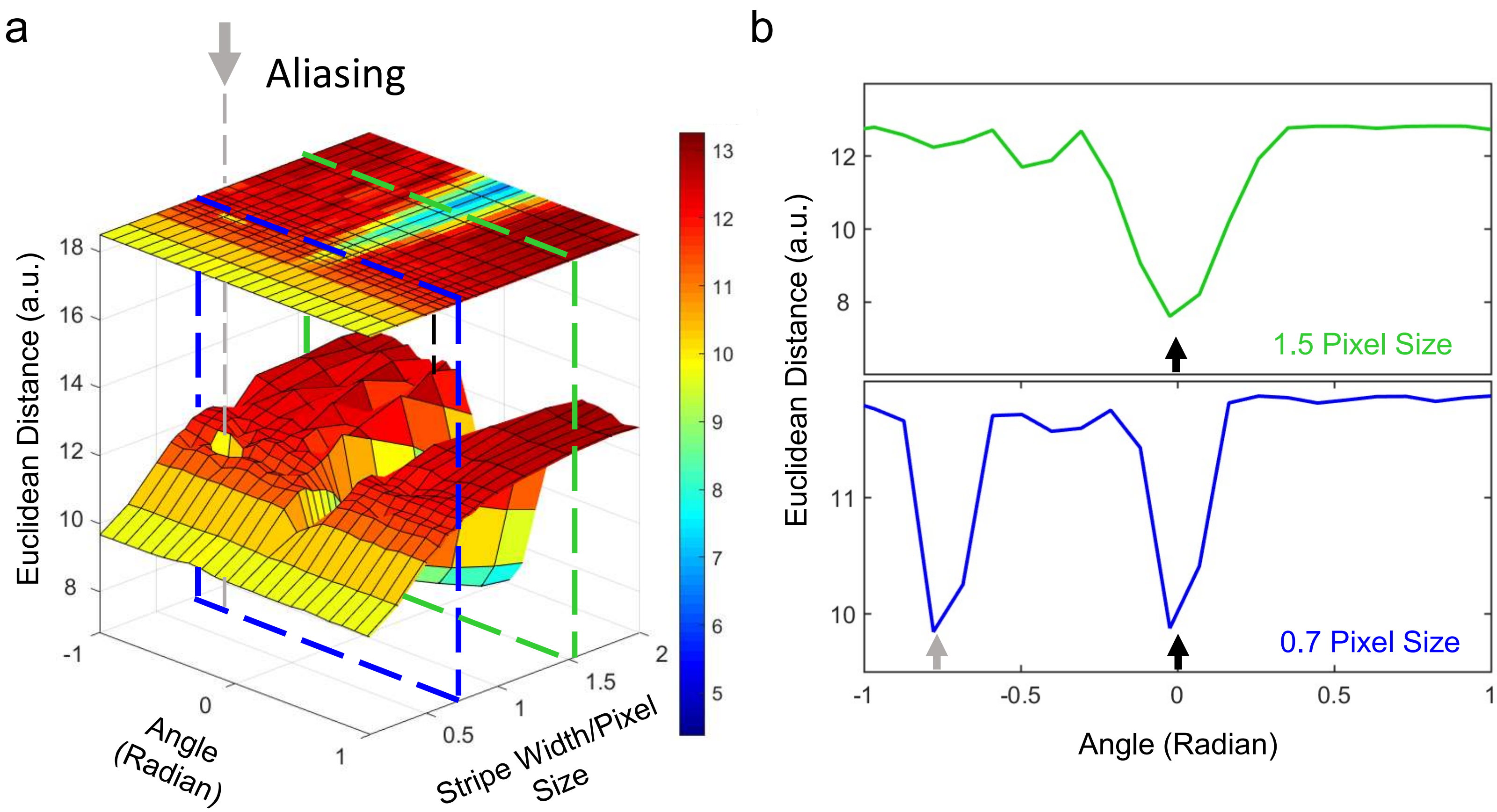

Supplement: supplementary [file NIHMS1575944-supplement-supplementary.pdf]
